# Combination of Epigallocatechin-3-Gallate and Tramiprosate Prevent Accumulation of Intracellular Aβ and Dysfunctional Autophagy–Lysosomal Pathway at Earliest Stage of Transdifferentiation of Mesenchymal Stromal Cells into PSEN1 E280A Cholinergic-like Neurons

**DOI:** 10.3390/ijms26083756

**Published:** 2025-04-16

**Authors:** Viviana Soto-Mercado, Miguel Mendivil-Perez, Marlene Jimenez-Del-Rio, Carlos Velez-Pardo

**Affiliations:** 1 Neuroscience Research Group, Institute of Medical Research, Faculty of Medicine, University Research Headquarters, Calle 62#52-59, Building 1, Laboratory 411/412, Medellin 050010, Colombia; viviana.soto@udea.edu.co (V.S.-M.); marlene.jimenez@udea.edu.co (M.J.-D.-R.); 2 Neuroscience Research Group, Faculty of Nursing, University Research Headquarters, Calle 62#52-59, Building 1, Laboratory 411/412, Medellin 050010, Colombia; miguel.mendivil@udea.edu.co

**Keywords:** Alzheimer, amyloid-beta, autophagy, cholinergic, e280a, epigallocatechin-3-gallate, mutation, tramiprosate

## Abstract

Familial Alzheimer’s disease (FAD) caused by presenilin 1 (PSEN1) E280A induces the aberrant accumulation of intracellular Aβ (iAβ) in cholinergic-like neurons (ChLNs). How early iAβ accumulates in the development of ChLNs is still unknown. Consequently, the timing of appropriate therapeutic approaches against FAD is unclear. To determine the earliest iAβ in PSEN1 E280A ChLNs, flow cytometry and immunofluorescence microscopy were used to follow the development of menstrual mesenchymal stromal cells (MenSCs) into ChLNs (proliferation marker Ki67, cluster of differentiation 73 (CD73), neuronal nuclei (NeuN) marker, choline acetyl transferase (ChAT)), the kinetics of iAβ accumulation, and the simultaneous evaluation of other associated markers (e.g., DJ-1C106-SO_3_; lysosomes; phosphatidylethanolamine-conjugated microtubule-associated protein 1A/1B light chain 3, LC3-II; cleaved caspase 3 (CC3)) at 0, 1, 3, 5, and 7 days. To reverse the PSEN1 E280A phenotype, we used rapamycin (RAP), verubecestat (VER), compound E (CE), epigallocatechin-3-gallate (EGCG), and tramiprosate (TM) in WT and mutant ChLNs. We found that PSEN1 E280A did not induce significant differences in the NeuN marker and ChAT in MenSCs transitioning to ChLNs. The iAβ accumulates at the earliest cholinergic developmental stage from day 0 (18%, at MenSCs stage) to day 7 (46%, at ChLNs stage), i.e., iAβ increased +156% in mutant compared to WT cells (1–6%). A significant increase in DJ-1C106-SO_3_ occurs only at day 7 (+250%). While neither CC3 (0–1%) nor lysosomes were different between WT and mutant cells at any time point, a stepwise increase in autophagosome accumulation was observed from day 3 (15%) to day 7 (79%), i.e., +427%, in mutant cells. While neither RAP, VER, nor CE was able to completely reduce all PSEN1 E280A-induced markers in ChLNs, the combination of EGCG and TM was more effective in removing these markers than EGCG and TM alone in PSEN1 E280A ChLNs. Given that this investigation is based on a single menstrual blood sample from WT and PSEN1 E280A, our results should be considered exploratory. Larger sample sizes are needed.

## 1. Introduction

Alzheimer’s disease (AD) is a progressive brain disorder that leads to dementia, which includes memory loss, confusion, thinking difficulties, and changes in language, behavior, and personality (https://www.alz.org/, accessed on 8 April 2025). It is estimated that AD accounts for at least 60–70% of all cases of dementia worldwide [1] and is a major global macroeconomic burden [2]. According to the onset of the disease, AD can be classified as early-onset (familial AD, <60 years of age) or late-onset (sporadic AD, >65 years of age). Although FAD is a subcategory of AD [3,4] with a prevalence of only 5% of AD cases, it has provided invaluable insights into the pathogenesis of sporadic AD. Indeed, FAD is caused by highly penetrant pathogenic mutations in the presenilin 1 (*PSEN1*), presenilin 2 (*PSEN2*), and amyloid precursor protein (*APP*) genes (https://www.alzforum.org/mutations, accessed on 10 March 2025) [5]. The PSEN forms a protease complex (PSEN/γ secretase) [6] responsible for the proteolytic processing of APP, where this type I transmembrane protein can be processed through a non-amyloidogenic and an amyloidogenic pathway. The former pathway involves the cleavage of APP by α- and γ-secretases, resulting in a long-secreted form of APP (sAPPα) and C-terminal fragments (CTF 83, p3, and AICD50). The amyloidogenic pathway involves the cleavage of APP by β- and γ-secretases, culminating in the production of a long-secreted form of APP (sAPPβ), C-terminal fragments (CTF99 and CTF89), and Aβ fragments, predominantly peptide Aβ40. However, the loss of PSEN1 function has been shown to lead to an aberrant increase in the generation of Aβ42 [7] by molecular mechanisms that are not yet fully understood [8,9]. It has been shown that Aβ is produced from CTF99 by PSEN/γ-secretase within acidic compartments, such as lysosomes and late endosomes in living neurons [10]. Furthermore, these authors have shown that intracellular Aβ (iAβ) is significantly accumulated in the same subcellular compartments. Consequently, PSEN1 has been identified as a critical factor for efficient proteolysis via the autophagy–lysosome pathway (ALP) in a γ-secretase-independent manner [11,12]. These observations have led to the hypothesis that alterations in ALP may play an important role in the pathogenesis of AD [13]. These observations may explain why iAβ accumulation may be the earliest pathological finding in in vivo and in vitro models of FAD [14,15,16,17,18,19,20]. However, the timing of the appearance of iAβ in human neurons and the extent to which ALP contributes to neuronal dysfunction in FAD remain to be fully elucidated. Addressing these questions is imperative, as it may facilitate the identification of the pharmacological window within which FAD should be treated [21]. Furthermore, it would be highly informative to determine whether the use of rapamycin (RAP), verubecestat (VER), and compound E (CE), which are autophagy-inducing agents [22,23], β-secretase (BACE1) inhibitors [24], and γ-secretase inhibitors [25], respectively, could hold promise for the prevention of FAD [26,27,28,29] if used early enough in PSEN1 E280A patients. Addressing these questions may also facilitate the determination of whether FAD can be mechanistically classified as a developmental disorder [30]. Overall, mutations in PSEN1 could act via the PSEN1/γ-secretase-dependent pathway, i.e., the abnormal accumulation of iAβ-induced cell death [31], the PSEN1/γ-secretase-independent pathway, i.e., the alteration of ALP [32], or both pathways. However, the lack of available data precludes the ability to distinguish between these pathways.

To date, at least 350 PSEN1 variants have been associated with FAD (https://www.alzforum.org/mutations/psen-1, accessed on 10 March 2025). Notably, in 1995, a pathologic missense mutation in PSEN1, located on chromosome 14 at 73664808 A > C (codon change: GAA to GCA, exon 8), was reported to consist of a glutamic acid (E) to alanine (A) change at codon 280 (p.E280A) [33]. The age and geographic origin of E280A led to the conclusion that it was the result of a single founder effect from the time of the Spanish conquistadors, who began colonizing Colombia in the early 16th century [34]. PSEN1 E280A, also known as the “Paisa mutation,” has been identified as the primary cause of familial early-onset Alzheimer’s disease (FAD) in the state of Antioquia, Colombia [35]. Despite ongoing research efforts to prevent PSEN1 E280A-induced FAD (e.g., clinicaltrials.gov NCT01998841, accessed on 10 March 2025), there is currently no effective treatment to delay, prevent, or cure FAD. This underscores the urgent need for the development of novel therapeutic interventions.

**Figure 1:**
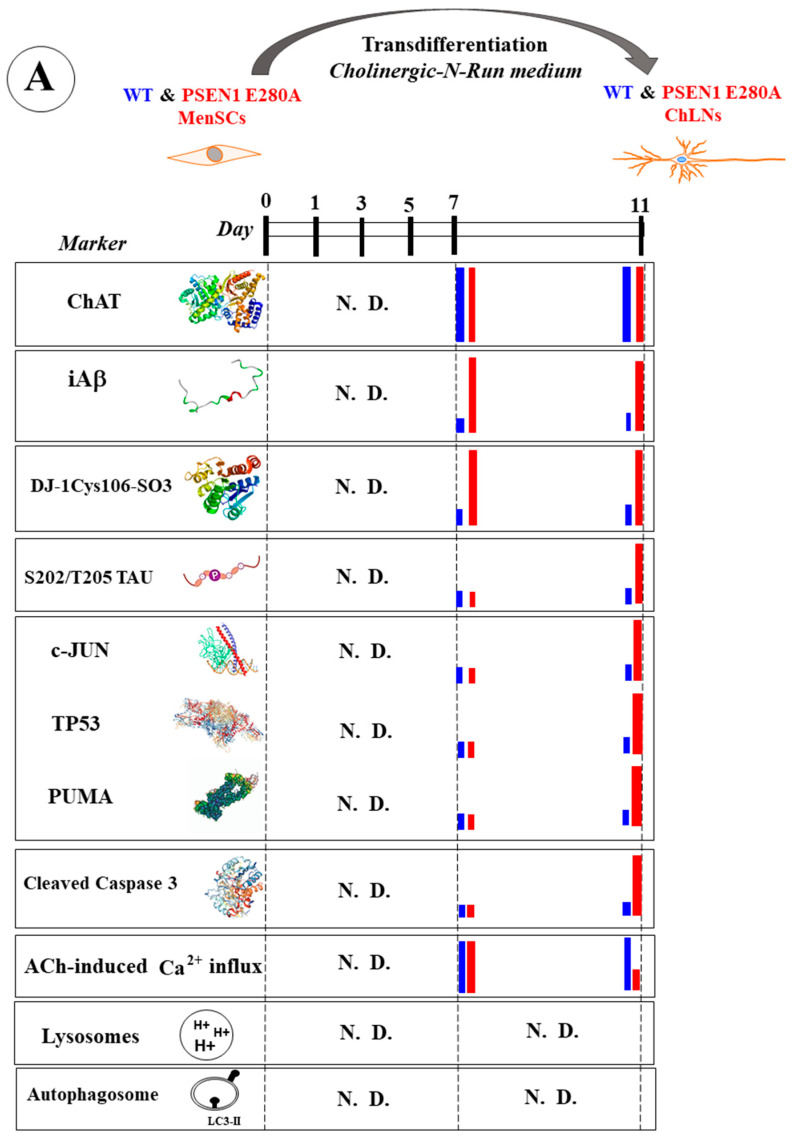

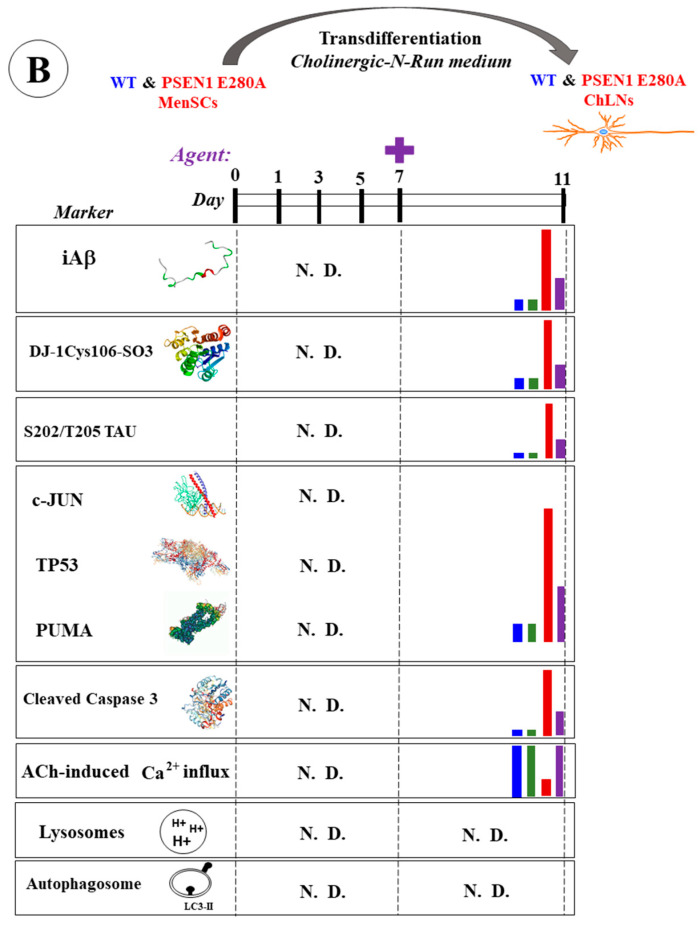
Schematic representation of wild-type (WT) and PSEN1 E280A menstrual stromal cell (MenSC) transdifferentiation into cholinergic-like neurons (ChLNs), appearance of neuropathologic markers, and effects of agents. (**A**) WT and PSEN1 ChLNs MenSCs were transdifferentiated into WT and PSEN1 E280A ChLNs in cholinergic N-Run (Ch-N-R) medium for 7 days and cultured in regular culture medium (RCm) for an additional 4 days. On days 7 and 11, markers (as listed) were evaluated in WT (blue bar) and PSEN1 E280A (red bar). N.D. = not determined. (**B**) WT and PSEN1 ChLNs were exposed to agents (purple letters represent reagent SP600125, CP55940, minocycline, sildenafil, epigallocatechin-3-gallate, melatonin) on day 7 (plus sign) and left in culture for 4 additional days. On day 11, untreated WT ChLNs (blue bar), ChLNs treated cells with agent only (green bar), untreated PSEN1 E280A ChLNs (red bar), or PSEN1 E280A ChLNs treated with agent (purple bar) were analyzed for different markers (as listed). N.D. = not determined.

Several data suggest that neurodegeneration within the basal forebrain cholinergic cell group Ch4 of the nucleus basalis of Meynert (NbM), which projects to the cortex and amygdala, precedes and predicts both entorhinal and memory deficits [36]. Indeed, the preferential accumulation of Aβ and p-TAU has been shown in the NbM [37,38], which may contribute to the structural degeneration of the NbM [39]. In an effort to recapitulate the neuropathology of FAD in vitro, we established PSEN1 E280A cholinergic-like neurons (ChLNs) derived from umbilical cord Wharton’s jelly mesenchymal stromal cells (WJ-MSCs) using cholinergic-N-run medium (Ch-N-Rm) [40]. On day 7 of the transdifferentiation process, mutant ChLNs exhibited a pronounced accumulation of iAβ42 and oxidized DJ-1 (i.e., DJ-1C106-SO_3_), indicative of oxidative stress (OS), without any evidence of cell death [41]. However, by day 11, cholinergic mutant cells exhibited abnormal phosphorylation of the protein TAU (at S202/T205), apoptogenic-positive markers, such as tumor protein 53 (TP53), p-S63/S73 c-JUN, the p53-upregulated modulator of apoptosis (PUMA), cleaved caspase 3 (CC3), the loss of mitochondrial membrane potential (ΔΨm), and dysfunctional acetylcholine (ACh)-induced Ca^2+^ ion influx [41] (Figure 1A). These observations suggest that iAβ not only induces p-TAU [42,43] but also triggers a series of signals that lead to neuronal dysfunction. The c-Jun N-terminal kinase (JNK) inhibitor SP600125 has been shown to reduce p-TAU [42]. Indeed, the accumulation of iAβ is associated with OS, and both phenomena precede p-TAU, apoptosis, and cholinergic dysfunction. Interestingly, in addition to the JNK inhibitor SP600125, the cannabinoid CP55940 [44], minocycline [45], sildenafil [46], and epigallocatechin-3-gallate (EGCG) alone [47] or in combination with melatonin [48], added at day 7 of transdifferentiation, protected PSEN1 E280A ChLNs against Aβ toxic effects until day 11 (Figure 1B, all reagent mentioned are collectively referred as the *Agent*, letter in purple). However, further investigation is needed to determine the following: (i) whether the production and accumulation of iAβ starts at day 0 of the transdifferentiation of MenSCs towards the PSEN1 E280A cholinergic lineage (Figure 1A, N.D. = not determined); (ii) whether iAβ affects ALP or induces apoptosis at the MenSCs-ChLNs transition (Figure 1A, N.D.); or (iii) whether EGCG and tramiprosate (TM)—two anti-antioxidant/anti-Aβ aggregation agents [49,50,51], RAP, VER, or CE—prevent iAβ-induced cell changes in MenSCs transitioning to PSEN1 E280A ChLNs (Figure 1B, N.D.).

To gain insight into these issues, we sought to investigate the earliest pathological FAD markers, such as iAβ and oxidized DJ-1, in human-derived cells. To accomplish this, we obtained menstrual stromal cell (MenSCs)-derived WT and PSEN1 E280A ChLNs as a valid model of FAD [52] for up to 7 days of culture in Ch-N-Rm. Using flow cytometry and fluorescence microscopy techniques, we examined the accumulation of iAβ42, the presence of DJ-1C106-SO_3_, and the appearance of phosphatidylethanolamine-conjugated microtubule-associated protein 1A/1B light chain 3 (hereafter referred to as LC3-II)—a marker of autophagosome development in ALP [53]—and the detection of pH-lysosomal vesicles with LysoTracker^®^—a dye that stains acidic compartments [54]. Proteinopathy, OS, and autophagy were assessed on days 1, 3, 5, and 7. In addition, ChLNs were exposed to the BACE-1 and γ secretase inhibitors VER and CE, the autophagy inducer RAP, and the autophagy inhibitor bafilomycin A1 as an internal control (BAF) [55] until day 7. It has been theorized that EGCG induces neuroprotection in AD through multiple mechanisms, including a reduction in OS, the prevention of Aβ aggregation, the alteration of cell signaling pathways, the promotion of autophagy, and the enhancement of mitochondrial activity [47,48,56]. TM has shown anti-apoptotic, antioxidant, and anti-amyloid properties in PSEN1 I416T ChLNs [51] and has been used as a prodrug in clinical trials for AD [57]. Given these chemical properties, PSEN1 E280A MenSCs that had undergone transdifferentiation into a cholinergic lineage were subjected to treatment with EGCG and TM, either individually or in combination, from day 0 to day 7.

## 2. Results

### 2.1. PSEN1 E280A Delays the Transdifferentiation of MenSCs to the Cholinergic Neuron Lineage

**Figure 2:**
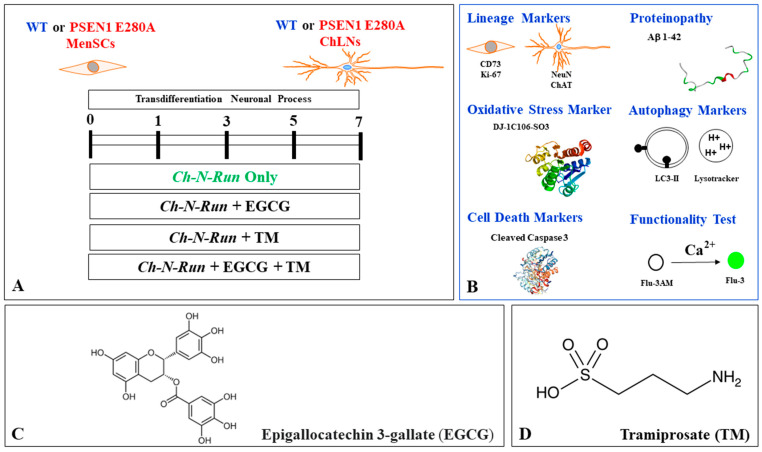
Schematic diagram of experimental procedure, biochemical analysis, chemical structure, and molecular formula of epigallocatechin-3-gallate (EGCG) and tramiprosate (TM). (**A**) PSEN1 E280A menstrual mesenchymal stromal cells (MenSCs) cultured in cholinergic-N-Run (Ch-N-R) medium for 0, 1, 3, 5, and 7 days transdifferentiated into cholinergic-like neurons (ChLNs). Reagents were added at day 0 (start transdifferentiation) and left in culture up to day 7. (**B**) ChLNs were then evaluated for lineage markers, including cluster of differentiation (CD) 73, proliferation marker Ki-67, neuronal nuclear antigen (NeuN), cholinergic lineage marker choline acetyltransferase (ChAT); proteinopathy of disease, accumulation of intracellular Aβ, oxidized DJ-1C106-SO_3_ as indication of generation of hydrogen peroxide (H_2_O_2_), oxidative stress (OS); autophagy markers LC3-phosphatidylethanolamine (LC3-II) conjugate as an indication of the presence of autophagosomes and LysoTracker as an indication of the presence of lysosomes; cell death markers (e.g., cleaved caspase 3 (CC3)); and functionality test acetylcholine-induced transient Ca^2+^ influx. (**C**) Epigallocatechin-3-gallate (EGCG); C22H18O11; [(2R,3R)-5,7-dihydroxy-2-(3,4,5-trihydroxyphenyl)-3,4-dihydro-2H-chromen-3-yl]3,4,5-trihydroxybenzoate; (**D**) tramiprosate (TM) or homotaurine; 3-aminopropane-1-sulfonic acid, C3H9NO3S.

**Figure 3:**
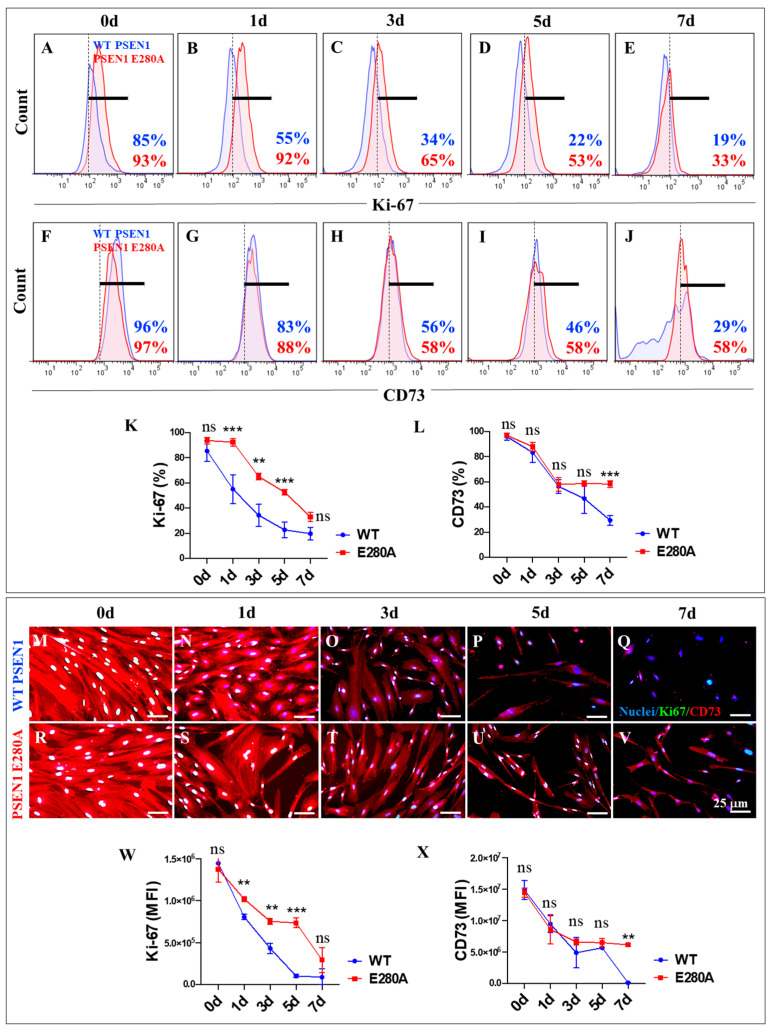
Flow cytometry and immunofluorescence analysis of proliferation marker Ki67 and cluster of differentiation 73 (CD73) during the process of transdifferentiation of menstrual mesenchymal stromal cells (MenSCs) into cholinergic-like neurons (ChLNs). Representative histograms showing Ki67 (**A**–**E**) and CD73 (**F**–**J**) flow cytometry analysis performed in wild-type (WT) PSEN1 (blue histograms) and E280A (red histograms) MenSCs after 0 (**A**,**F**), 1 (**B**,**G**), 3 (**C**,**H**), 5 (**D**,**I**), and 7 (**E**,**J**) days of culture in Ch-N-Rm. (**K**,**L**) Bar charts represent the quantitative analysis of the positive population. Representative images showing Ki67 (green) and CD73 (red) double-stained WT PSEN1 (M-Q) and E280A (**R**–**V**) MenSCs after 0 (**M**,**R**), 1 (**N**,**S**), 3 (**O**,**T**), 5 (**P**,**U**), and 7 (**Q**,**V**) days of culture in Ch-N-Rm. Nuclei were stained with Hoechst dye (blue; **M**–**V**). (**W**,**X**) Bar charts represent the mean fluorescent intensity (MFI) quantification of images obtained by immunofluorescence analysis. The histograms and images represent one out of three independent experiments. Significant values were determined by two-way ANOVA with a Tukey post hoc test; ** *p* < 0.01, *** *p* < 0.001 intergroup comparison; n.s. = not significant. Image magnification 20×.

Previous studies have shown that both wild-type (WT) and PSEN1 E280A MenSCs transdifferentiate into cholinergic neurons (ChLNs) under Ch-N-Run medium for 7 days, with no apparent effect of the mutation on this process [41]. Therefore, we wanted to determine whether the mutation alters the proliferation or expression of neuronal markers in the transdifferentiation process, following the decrease in the proliferative marker Ki-67, the disappearance of the mesenchymal marker CD73, the appearance of the nuclear neuronal marker NeuN, and the presence of the specific cholinergic lineage marker ChAT from day 0 to day 7, according to the protocol described in Figure 2A (letters in green color) and 2B (lineage markers). As shown in Figure 3, the Ki-67 (Figure 3A–E) and CD73 (Figure 3F–J) markers showed a time-dependent decrease in WT (Figure 3K–L), while the markers gradually decreased in PSEN1 E280A cells. Notably, the cellular proliferative capacity of mutant cells was significantly different from that of WT cells (Figure 3K), and PSEN1 E280A cells significantly retained cell lineage immaturity until day 7 (Figure 3L). Similar observations were obtained by fluorescence microscopy analysis (Figure 3M–X).

**Figure 4:**
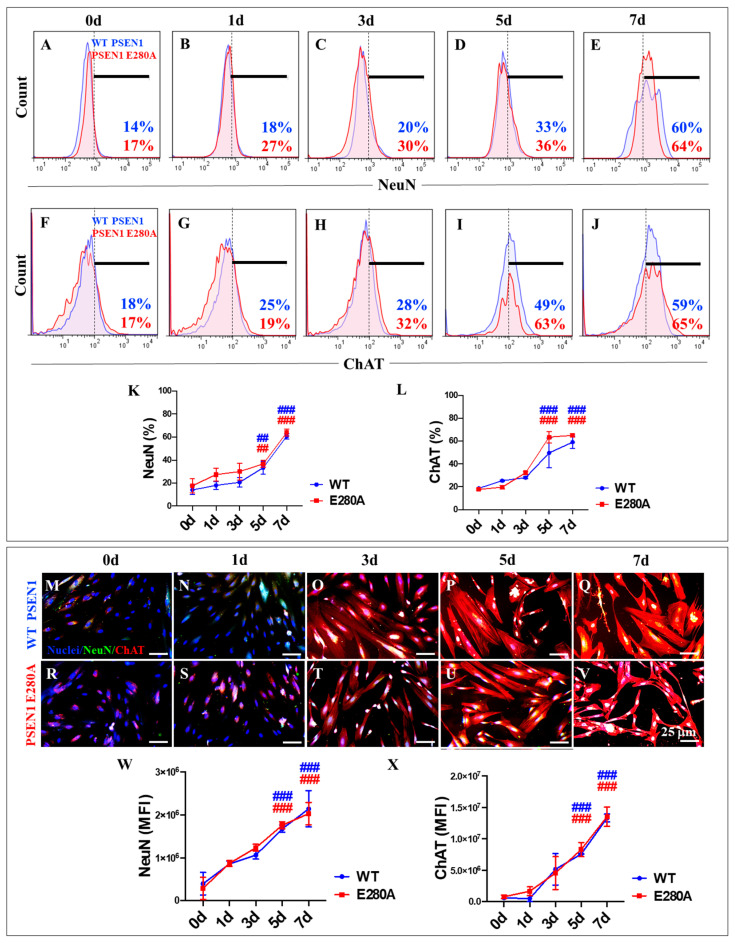
Flow cytometry and immunofluorescence analysis of neuronal nuclei (NeuN) and choline acetyl transferase (ChAT) during the process of transdifferentiation of menstrual mesenchymal stromal cells (MenSCs) into cholinergic-like neurons (ChLNs). Representative histograms showing NeuN (**A**–**E**) and ChAT (**F**–**J**) flow cytometry analysis performed in WT PSEN1 (blue histograms) and E280A (red histograms) ChLNs after 0 (**A**,**F**), 1 (**B**,**G**), 3 (**C**,**H**), 5 (**D**,**I**), and 7 (**E**,**J**) days of culture in Ch-N-Rm. (**K**,**L**) Bar charts represent the quantitative analysis of the positive population. Representative images showing NeuN (green) and ChAT (red) double-stained WT PSEN1 (**M**–**Q**) and E280A (**R**–**V**) ChLNs after 0 (**M**,**R**), 1 (**N**,**S**), 3 (**O**,**T**), 5 (**P**,**U**), and 7 (**Q**,**V**) days of culture in Ch-N-Rm. Nuclei were stained with Hoechst dye (blue; **M**–**V**). (**W**,**X**) Bar charts represent the mean fluorescent intensity (MFI) quantification of images obtained by immunofluorescence analysis. The histograms and images represent one out of three independent experiments. Significant values were determined by two-way ANOVA with a Tukey post hoc test; ## *p* < 0.01, ### *p* < 0.001 intragroup comparison vs. 0d. Image magnification 20×.

Furthermore, our statistical analysis revealed no significant differences in the expression levels of the neuronal marker NeuN (Figure 4A–E) and the cholinergic marker ChAT (Figure 4F–J) in WT and PSEN1 E280A cells during the transdifferentiation process (Figure 4K,L). These results were confirmed by fluorescence microscopy analysis (Figure 4M,X).

### 2.2. PSEN1 E280A Induces a Steady, Time-Dependent Increase in the Accumulation of Intracellular Aβ (iAβ) in Cholinergic-like Neurons (ChLNs)

**Figure 5:**
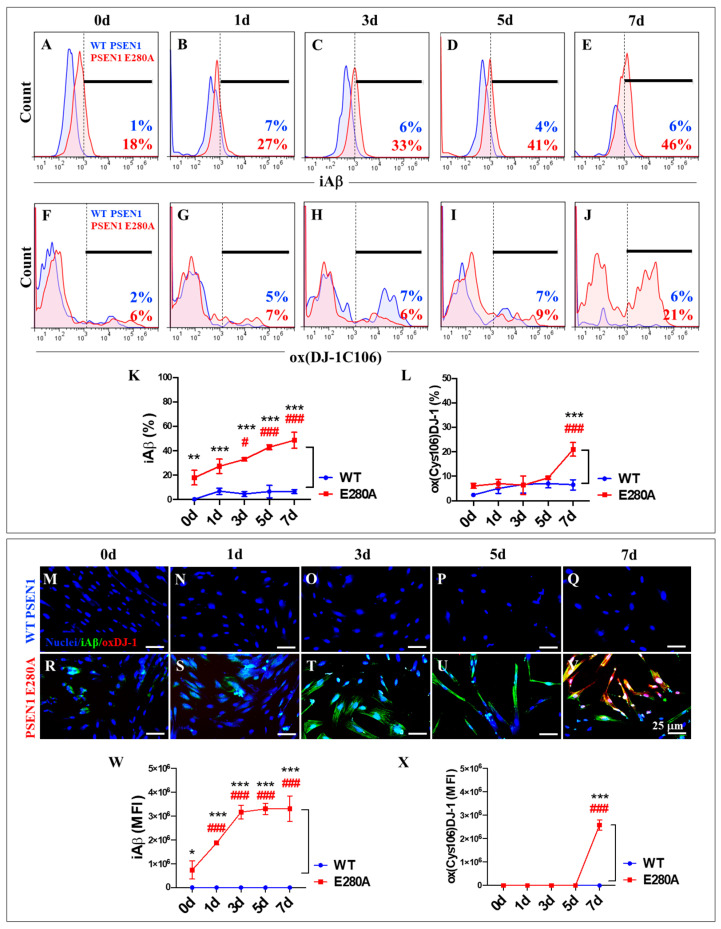
Flow cytometry and immunofluorescence analysis of intracellular Aβ42 and oxidized DJ-1 during the process of transdifferentiation of menstrual mesenchymal stromal cells (MenSCs) into cholinergic-like neurons (ChLNs). Representative histograms showing iAβ42 (**A**–**E**) and oxDJ-1 C106 (**F**–**J**) flow cytometry analysis performed in WT PSEN1 (blue histograms) and E280A (red histograms) ChLNs after 0 (**A**,**F**), 1 (**B**,**G**), 3 (**C**,**H**), 5 (**D**,**I**), and 7 (**E**,**J**) days of culture in Ch-N-Rm. (**K**,**L**) Bar charts represent the quantitative analysis of the positive population. Representative images showing iAβ42 (green) and oxDJ-1 C106 (red) double-stained WT PSEN1 (**M**–**Q**) and E280A (**R**–**V**) ChLNs after 0 (**M**,**R**), 1 (**N**,**S**), 3 (**O**,**T**), 5 (**P**,**U**), and 7 (**Q**,**V**) days of culture in Ch-N-Rm. Nuclei were stained with Hoechst dye (blue; **M**–**V**). (**W**,**X**) Bar charts represent the mean fluorescent intensity (MFI) quantification of images obtained by immunofluorescence analysis. The histograms and images represent one out of three independent experiments. Significant values were determined by two-way ANOVA with a Tukey post hoc test; # *p* < 0.05, ### *p* < 0.001 intragroup comparison vs. 0d. * *p* < 0.05, ** *p* < 0.01, *** *p* < 0.001 intergroup comparison. Image magnification 20×.

Next, the time course of iAβ accumulation and the presence of oxDJ-1 were examined as evidence of oxidative stress (OS) from day 0 to day 7 in the transdifferentiation of MenSCs into ChLNs. To this end, wild-type (WT) and PSEN1 E280A MenSCs were cultured in Ch-N-Run medium (Figure 2A) and analyzed for proteinopathy and oxidized DJ-1 markers on days 0, 1, 3, 5, and 7 (Figure 2B). As shown in Figure 5, flow cytometry analysis revealed that wild-type PSEN1 cells exhibited a consistent low basal accumulation of iAβ over the course of the study, whereas mutant PSEN1 cells induced a steady, time-dependent increase in iAβ (Figure 5A–E,K). Overall, PSEN1 E280A increased iAβ accumulation by +155% from day 0 to day 7. Notably, a significant accumulation of iAβ (+1700%) was detected in PSEN1 E280A MenSCs at day 0 compared to WT MenSCs (Figure 5A,K). We found that both WT and PSEN1 E280A cells exhibited a basal level of oxidized DJ-1 up to day 5 (Figure 5F–J,L), whereas a sudden increase of +250% in oxidized DJ-1 was detected in PSEN1 E280A ChLNs at day 7 (Figure 5J,L). These observations were confirmed by the subsequent analysis of the samples by fluorescence microscopy (Figure 5M–X).

### 2.3. PSEN1 E280A Induces a Steady, Time-Dependent Increase in the Accumulation of Autophagosomes, but Not Lysosomes, in the Absence of Cleaved Caspase 3 (CC3) in Cholinergic-like Neurons (ChLNs)

**Figure 6:**
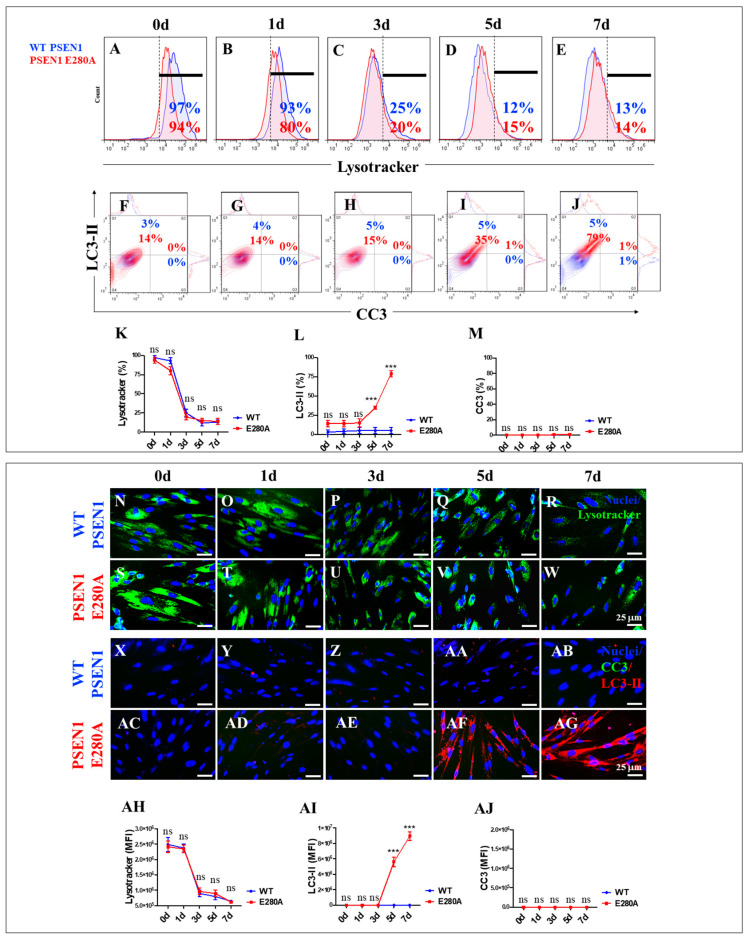
Flow cytometry and fluorescence microscopy analysis of lysosome acidification, phosphatidylethanolamine-conjugated microtubule-associated protein 1A/1B light chain 3 (LC3-II) accumulation, and cleaved caspase 3 (CC3) reactivity during the process of transdifferentiation of menstrual mesenchymal stromal cells (MenSCs) into cholinergic-like neurons (ChLNs). Representative histograms (**A**–**E**) showing LysoTracker flow cytometry analysis performed in WT PSEN1 (blue histograms) and E280A (red histograms) ChLNs after 0 (**A**), 1 (**B**), 3 (**C**), 5 (**D**), and 7 (**E**) days of culture in Ch-N-Rm. Representative contour histograms showing LC3-II (y axis) and CC3 (x axis). (**F**–**J**) Flow cytometry double analysis performed in WT PSEN1 (blue contour) and E280A (red contour) ChLNs after 0 (**F**), 1 (**G**), 3 (**H**), 5 (**I**), and 7 (**J**) days of culture in Ch-N-Rm. (**K**–**M**) Bar charts represent the quantitative analysis of the positive LysoTracker (**K**), LC3-II (**L**), and CC3 (**M**) population. Representative images showing LysoTracker (green)-positive WT PSEN1 (**N**–**R**) and E280A (**S**–**W**) ChLNs after 0 (**N**,**S**), 1 (**O**,**T**), 3 (**P**,**U**), 5 (**Q**,**V**), and 7 (**R**,**W**) days of culture in Ch-N-Rm. Nuclei were stained with Hoechst dye (blue; **N**–**W**). Representative images showing CC3 (green) and LC3-II (red) double-stained WT PSEN1 (**X**–**AB**) and E280A (**AC**–**AG**) ChLNs after 0 (**X**,**AC**), 1 (**Y**,**AD**), 3 (**Z**,**AE**), 5 (**AA**,**AF**), and 7 (**AB**,**AG**) days of culture in Ch-N-Rm. Nuclei were stained with Hoechst dye (blue; **X**–**AG**). (**AH**–**AJ**). Bar charts represent the mean fluorescent intensity (MFI) quantification of images obtained by immunofluorescence analysis. The histograms, contour histograms, and images represent one out of three independent experiments. Significant values were determined by two-way ANOVA with a Tukey post hoc test; *** *p* < 0.001 intergroup comparison. n.s. = not significant. Image magnification 20×.

The autophagy–lysosomal pathway (ALP) is thought to be impaired during the earliest intraneuronal stage of Alzheimer’s disease (AD) [58]. Given the ability to monitor ALP using markers such as LC3-II and the lysosomal marker LysoTracker^®^, we investigated the effect of PSEN1 E280A-induced iAβ on ALP in mutant ChLNs during their transdifferentiation from MenSCs to ChLNs. As shown in Figure 5, lysosomes decreased in a time-dependent manner in both WT and PSEN1 E280A cells (Figure 6A–E). The accumulation of lysosomes (>90% LysoTracker^®^-positive cells) decreased significantly from day 2 to day 3 by ~−76% and then remained almost constant until day 7 (Figure 6K). Conversely, autophagosome accumulation remained unperturbed until day 3 (3% and 14% LC3-II-positive cells in WT and PSEN1 E280A cells, respectively) but steadily increased by +133% and +426% on days 5 and 7, respectively, in PSEN1 E280A cells only (Figure 6F–J,L). No CC3-positive cells were detected at any time point in either WT PSEN1 or PSEN1 E280A cells (Figure 6F–J,M). Similar results were obtained by fluorescence microscopy analysis (Figure 6N–AJ).

### 2.4. Administration of Rapamycin (RAP) Does Not Alter the Substantial Accumulation of iAβ and Oxidized DJ-1, but Verubecestat (VER) and Compound E (CE) Moderately Reduce iAβ Only in PSEN1 E280A Cholinergic-like Neurons

**Figure 7:**
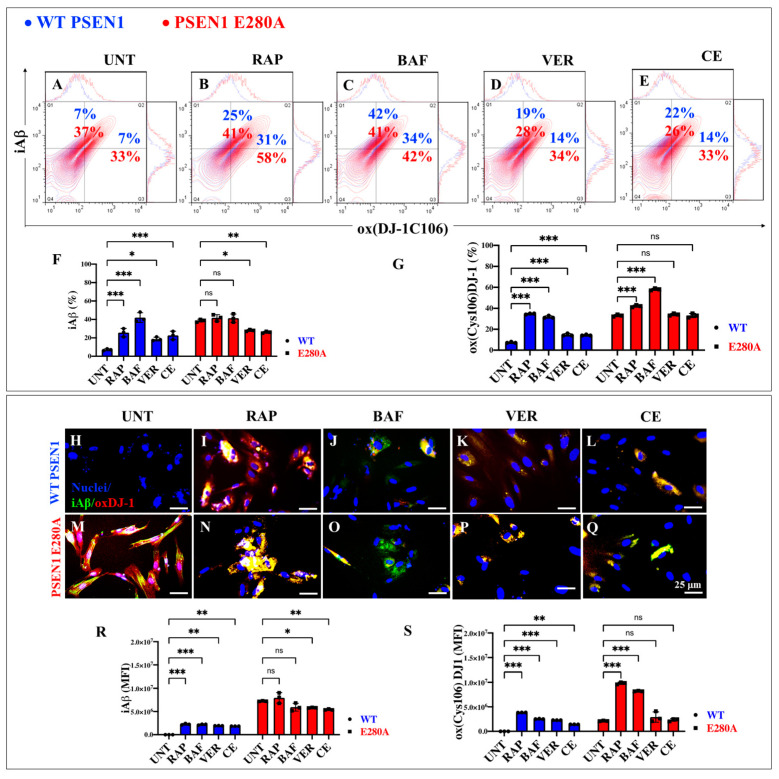
Flow cytometry and immunofluorescence analysis of intracellular Aβ42 and oxidized DJ-1 during the process of transdifferentiation of menstrual mesenchymal stromal cells (MenSCs) into cholinergic-like neurons (ChLNs) treated with rapamycin (RAP), bafilomycin (BAF), β-secretase inhibitor verubecestat (VER), and γ-secretase inhibitor compound E (CE). Representative contour histograms (**A**–**E**) showing iAβ42 (y axis) and oxDJ-1C106 (x axis) flow cytometry double analysis performed after 7 days in WT PSEN1 (blue contour) and E280A (red contour) ChLNs untreated (UNT) (**A**) or treated with 1 nM RAP (**B**), 1 nM BAF (**C**), 500 pM VER (**D**), and 500 pM CE (**E**). (**F**,**G**) Bar charts represent the quantitative analysis of the positive population. Representative images showing iAβ42 (green) and oxDJ-1 C106 (red) double-stained WT PSEN1 (**H**–**L**) and E280A (**M**–**Q**) ChLNs after 7 days untreated (**H**,**M**) or treated with 1 nM RAP (**I**,**N**), 1 nM BAF (**J**,**O**), 500 pM VER (**K**,**P**), and 500 pM CE (**L**,**Q**) days of culture in Ch-N-Rm. Nuclei were stained with Hoechst dye (blue; **H**–**Q**). (**R**,**S**) Bar charts represent the mean fluorescent intensity (MFI) quantification of images obtained by immunofluorescence analysis. The contour figures and images represent one out of three independent experiments. Significant values were determined by two-way ANOVA with a Tukey post hoc test; * *p* < 0.05, ** *p* < 0.01, *** *p* < 0.001 intergroup comparison. n.s. = not significant. Image magnification 20×.

In the context of exploring therapeutic targets against iAβ accumulation, RAP, the autophagy inhibitor bafilomycin A1 (BAF) used as a control, verubecestat (VER), and compound E (CE) were evaluated. WT and PSEN1 MenSCs were exposed to these reagents in Ch-N-Run media from day 0 to day 7 (Figure 2A). Flow cytometric analysis revealed that untreated PSEN1 E280A cells exhibited a significant increase in iAβ (+428%). Treatment with these reagents differentially affected both WT and mutant ChLNs. RAP, BAF, VER, and CE induced a significant increase in iAβ by +257% (Figure 7B), +500% (Figure 7C), +171% (Figure 7D), and +214% (Figure 7E), respectively, in WT ChLNs (Figure 7F). Similarly, an analysis of oXDJ-1 shows that PSEN1 E280A ChLNs intrinsically exhibited a significant increase in oXDJ-1 of +371% (Figure 7A) compared to WT ChLNs (Figure 7G). RAP, BAF, VER, and CE induced a significant increase in oxDJ-1 of +342% (Figure 7B), +385% (Figure 7C), +100% (Figure 7D), and 100% (Figure 7E), respectively, in treated WT ChLNs compared to untreated WT ChLNs (Figure 7G). When mutant ChLNs were treated with RAP and BAF reagents, the results showed that these reagents significantly increased oxDJ-1 by +76% (Figure 7B,G) and +27% (Figure 7C,G), respectively, in mutant cells, whereas VER and CE did not induce significant changes in oxDJ-1 in PSEN1 E280A (Figure 7G). Fluorescence microscopy analysis showed similar results (Figure 7H–S).

### 2.5. Administration of Rapamycin (RAP), Verubecestat (VER), and Compound E (CE) Induces Similar Levels of Lysosomal Acidification in PSEN1 E280A Cholinergic-like Neurons Comparable to Untreated or Treated WT Cholinergic-like Neurons

**Figure 8:**
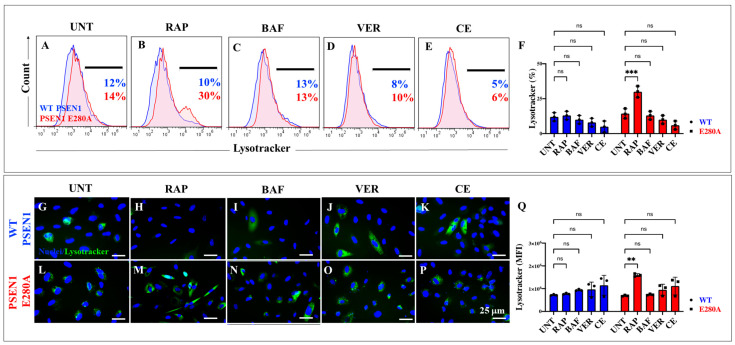
Flow cytometry and fluorescence microscopy analysis of lysosome acidification during the process of transdifferentiation of menstrual mesenchymal stromal cells (MenSCs) into cholinergic-like neurons (ChLNs) treated with rapamycin (RAP), bafilomycin (BAF), β-secretase inhibitor verubecestat (VER), and γ-secretase inhibitor compound E (CE). Representative histograms (**A**–**E**) showing LysoTracker flow cytometry analysis performed after 7 days in WT PSEN1 (blue histograms) and E280A (red histograms) ChLNs untreated (UNT) (**A**) or treated with 1 nM RAP (**B**), 1 nM BAF (**C**), 500 pM VER (**D**), and 500 pM CE (**E**). Bar chart showing the quantitative analysis of the LysoTracker^®^-positive population (**F**). Representative images showing LysoTracker (green)-positive WT PSEN1 (**G**–**K**) and E280A (**L**–**P**) ChLNs untreated (UNT) (**G,L**) or treated with 1 nM RAP (**H,M**), 1 nM BAF (**I,N**), 500 pM VER (**J,O**), and 500 pM CE (**K,P**). Nuclei were stained with Hoechst dye (blue; **G**–**P**). (**Q**) Represent the mean fluorescent intensity (MFI) quantification of images obtained by fluorescence microscopy analysis. The histograms and images represent one out of three independent experiments. Significant values were determined by two-way ANOVA with a Tukey post hoc test; ** *p* < 0.01, *** *p* < 0.001 intergroup comparison. n.s. = not significant. Image magnification 20×.

We next examined whether RAP, VER, and CE induced changes in lysosomal acidification in ChLNs. Figure 8A shows that there were no statistical differences between untreated WT and untreated PSEN1 E280A ChLNs (Figure 8A,F). Upon RAP treatment, PSEN1 E280A ChLNs significantly increased lysosomal acidification by +200% compared to WT ChLNs (Figure 8B,F). In contrast, BAF, VER, and CE show no significant changes in lysosomal signaling in both WT and mutant neuronal cells (Figure 8C–F). Similar observations were obtained by immunofluorescence microscopy analysis (Figure 8G–Q).

### 2.6. Administration of Rapamycin (RAP), Verubecestat (VER), and Compound E (CE) Induces Similar Levels of Autophagosome Accumulation (LC3-II) and Cleavage Caspase 3 (CC3) in PSEN1 E280A Cholinergic-like Neurons

**Figure 9:**
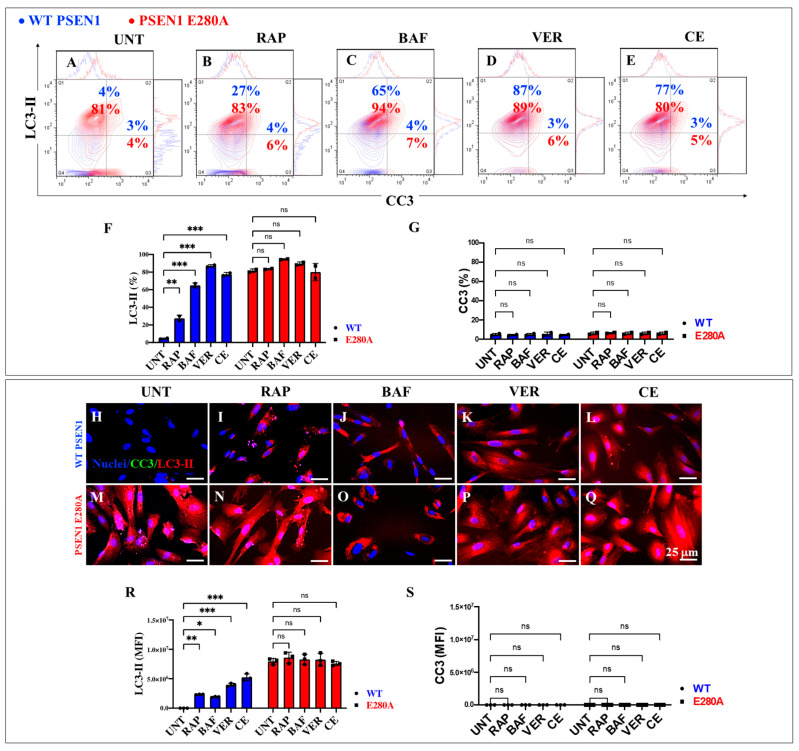
Flow cytometry and fluorescence microscopy analysis of LC3-II accumulation and cleaved caspase 3 (CC3) reactivity in cholinergic-like neurons (ChLNs) treated with rapamycin (RAP), bafilomycin (BAF), β-secretase inhibitor verubecestat (VER), and γ-secretase inhibitor compound E (CE). Representative contour histograms (**A**–**E**) showing LC3-II (y axis) and CC3 (x axis) flow cytometry analysis performed after 7 days in WT PSEN1 (blue contour) and E280A (red contour) ChLNs untreated (UNT) (**A**) or treated with 1 nM RAP (**B**), 1 nM BAF (**C**), 500 pM VER (**D**), and 500 pM CE (**E**). (**F**,**G**) Bar charts represent the quantitative analysis of the positive population. Representative images showing CC3 (green) and LC3-II (red) double-stained WT PSEN1 (**H**–**L**) and E280A (**M**–**Q**) ChLNs untreated (UNT) (**H**,**M**) or treated with 1 nM RAP (**I**,**N**), 1 nM BAF (**J**,**O**), 500 pM VER (**K**,**P**), and 500 pM CE (**L**,**Q**). Nuclei were stained with Hoechst dye (blue; **H**–**Q**). (**R**,**S**) Bar charts represent the mean fluorescent intensity (MFI) quantification of images obtained by immunofluorescence analysis. The contour figures and images represent one out of three independent experiments. Significant values were determined by two-way ANOVA with a Tukey post hoc test; * *p* < 0.05, ** *p* < 0.01 *** *p* < 0.001 intergroup comparison. n.s. = not significant. Image magnification 20×.

As an additional analysis of the autophagy system, we examined autophagosomes in WT and PSEN1 E280A ChLNs. Figure 9 shows that untreated mutant ChLNs exhibited an abnormal accumulation of autophagosomes (LC3-II-positive cells) by +1925% compared to untreated WT ChLNs (Figure 9A,F). RAP, BAF, VER, and CE differentially affected phagosome organelles in both WT and mutant cells. While RAP, BAF, VER, and CE increased phagosome accumulation in WT ChLNs by +575% (Figure 9B), +1525% (Figure 9C), +2075% (Figure 9D), and +1825% (Figure 9E), respectively (Figure 9F), no significant changes in autophagosome accumulation were observed in PSEN1 E280A ChLNs (Figure 9B–F). Notably, no significant changes in CC3 were observed in WT or PSEN1 E280A ChLNs in response to these reagents (Figure 9B–E,G). Similar results were obtained by fluorescence microscopy analysis (Figure 9H–S).

### 2.7. EGCG and TM Alone or in Combination Dramatically Reduce the Accumulation of iAβ Oxidized DJ-1 and the Accumulation of Autophagosomes in the Absence of Lysosomal Signal and CC3 in PSEN1 E280A ChLNs

**Figure 10:**
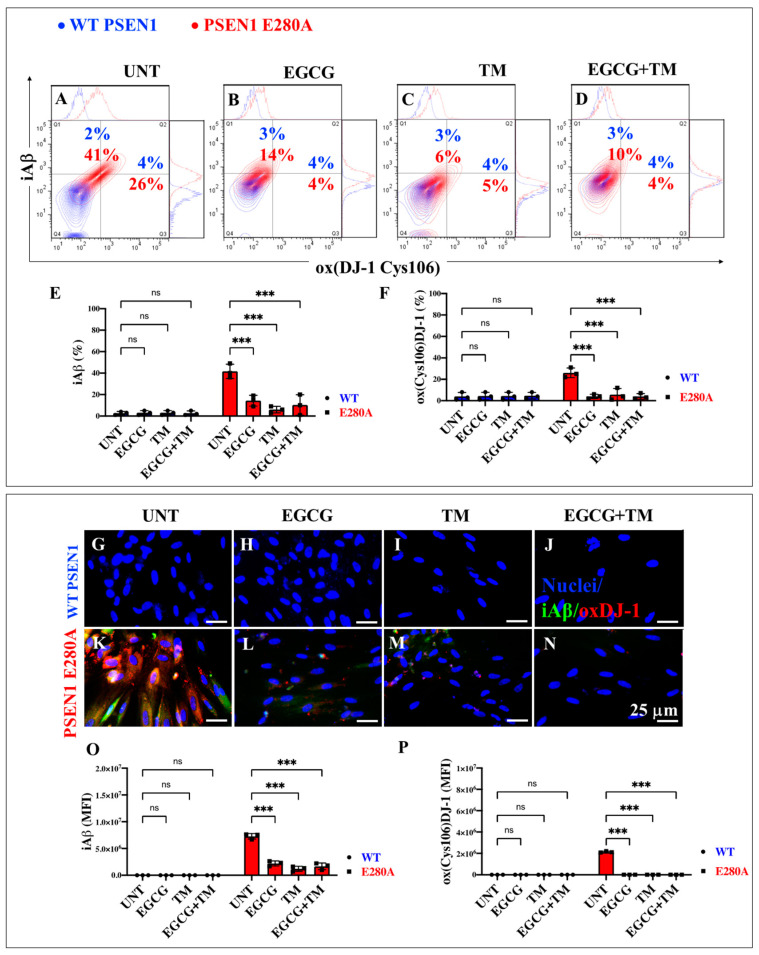
Flow cytometry and immunofluorescence analysis of intracellular Aβ42 and oxidized DJ-1 during the process of transdifferentiation of menstrual mesenchymal stromal cells (MenSCs) into cholinergic-like neurons (ChLNs) treated with (−)-epigallocatechin-3-gallate (EGCG) and tramiprosate (TM) alone or in combination. Representative contour histograms (**A**–**D**) showing iAβ42 and ox(DJ-1 C106) flow cytometry analysis performed after 7 days in WT PSEN1 (blue contour) and E280A (red contour) ChLNs untreated (UNT) (**A**) or treated with 25 μM EGCG (**B**) and 50 μM TM (**C**) alone or in combination (**D**). (**E**,**F**) Bar charts represent the quantitative analysis of the positive population. Representative images showing iAβ42 (green) and ox(DJ-1 C106) (red) double-stained WT PSEN1 (**G**–**J**) and E280A (**K**–**M**) ChLNs untreated (UNT) (**G**,**K**) or treated with 25 μM EGCG (**H**,**L**) and 50 μM TM (**I**,**M**) alone or in combination (**J**,**N**). (**O**,**P**) Bar charts represent the mean fluorescent intensity (MFI) quantification of images obtained by immunofluorescence analysis. The contour figures and images represent one out of three independent experiments. Significant values were determined by two-way ANOVA with a Tukey post hoc test; *** *p* < 0.001, intergroup comparison. n.s. = not significant. Image magnification 20×.

**Figure 11:**
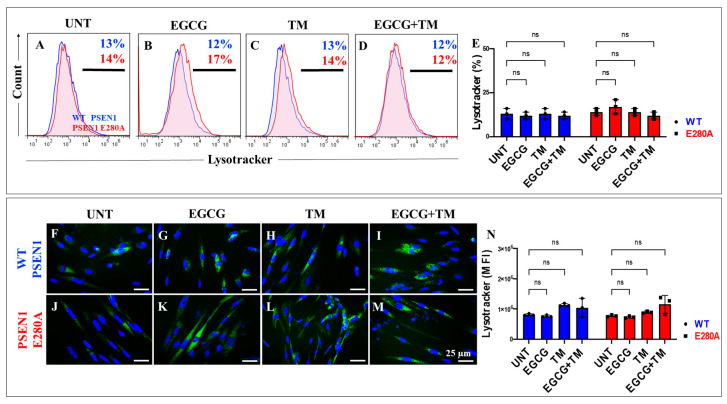
Flow cytometry and fluorescence microscopy analysis of lysosome acidification during the process of transdifferentiation of menstrual mesenchymal stromal cells (MenSCs) into cholinergic-like neurons (ChLNs) treated with (−)-epigallocatechin-3-gallate (EGCG) and tramiprosate (TM) alone or in combination. Representative histograms (**A**–**D**) showing LysoTracker flow cytometry analysis performed after 7 days in WT PSEN1 (blue histograms) and E280A (red histograms) ChLNs untreated (UNT) (**A**) or treated with 25 μM EGCG (**B**) and 50 μM TM (**C**) alone or in combination (**D**). (**E**) Bar chart represents the quantitative analysis of the positive population. Representative images showing LysoTracker (green)-positive WT PSEN1 (**F**–**I**) and E280A (**J**–**M**) ChLNs untreated (UNT) (**F**,**J**) or treated with EGCG (**G**,**K**) and TM (**H**,**L**) alone or in combination (**I**,**M**). Nuclei were stained with Hoechst dye (blue; **F**–**M**). (**N**) Bar chart represents the mean fluorescent intensity (MFI) quantification of images obtained by fluorescence microscopy analysis. The histograms and images represent one out of three independent experiments. Significant values were determined by two-way ANOVA with a Tukey post hoc test. n.s. = not significant. Image magnification 20×.

**Figure 12:**
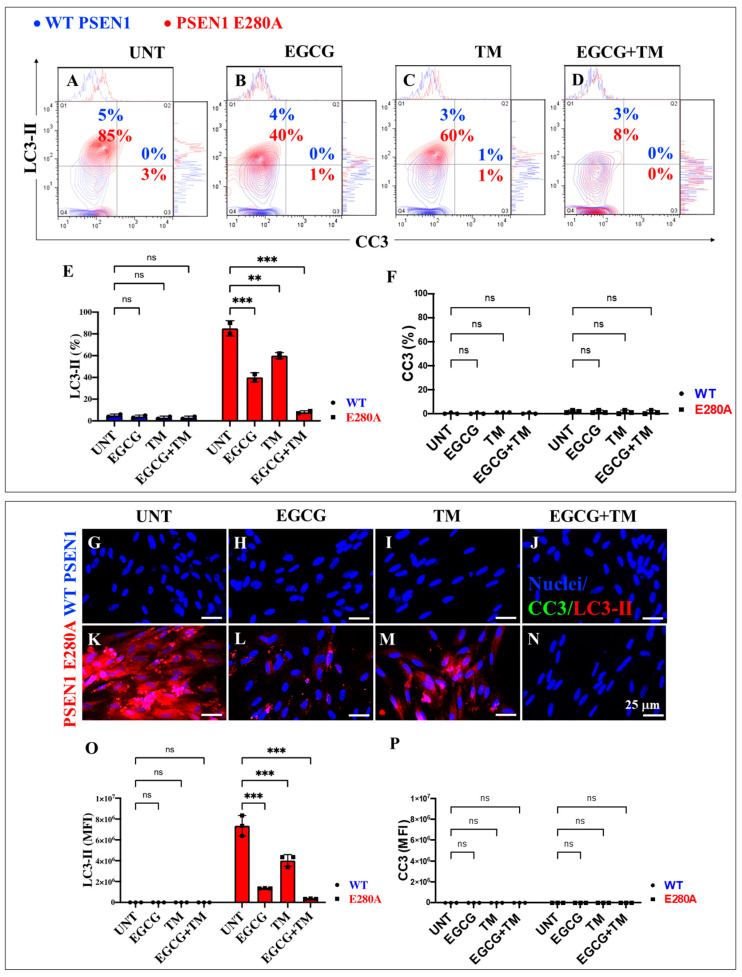
Flow cytometry and fluorescence microscopy analysis of LC3-II accumulation and cleaved caspase 3 (CC3) reactivity during the process of transdifferentiation of menstrual mesenchymal stromal cells (MenSCs) into cholinergic-like neurons (ChLNs) treated with (−)-epigallocatechin-3-gallate (EGCG) and tramiprosate (TM) alone or in combination. Representative contour histogram (**A**–**D**) showing LC3-II (y axis) and CC3 (x axis) flow cytometry analysis performed after 7 days in WT PSEN1 (blue contour) and E280A (red contour) ChLNs untreated (UNT) (**A**) or treated with 25 μM EGCG (**B**) and 50 μM TM (**C**) alone or in combination (**D**). (**E**,**F**) Bar charts represent the quantitative analysis of the positive population. Representative images showing CC3 (green) and LC3-II (red) double-stained WT PSEN1 (**G**–**J**) and E280A (**K**–**N**) ChLNs untreated (UNT) (**G**,**K**) or treated with 25 μM EGCG (**H**,**L**) and 50 μM TM (**I**,**M**) alone or in combination (**J**,**N**). Nuclei were stained with Hoechst dye (blue; **G**–**N**). (**O**,**P**) Bar charts represent the mean fluorescent intensity (MFI) quantification of images obtained by fluorescence microscopy analysis. The contour figures and images represent one out of three independent experiments. Significant values were determined by two-way ANOVA with a Tukey post hoc test, ** *p* < 0.01 *** *p* < 0.001 intergroup comparison. n.s. = not significant. Image magnification 20×.

The above observations prompted us to investigate whether the anti-amyloid and antioxidant agents epigallocatechin-3-gallate (EGCG, Figure 2C) and tramiprosate (TM, Figure 2D) alter the pathological phenotype in PSEN1 E280A ChLNs. To this end, wild-type (WT) and PSEN1 cells were exposed to EGCG and TM alone or in combination in Ch-N-Run media from day 0 (MenSCs) to day 7 (Figure 2A). As shown in the previous section, PSEN1 E280A ChLNs exhibited the characteristic FAD pathological phenotype within approximately 7 days of transdifferentiation, characterized by substantial accumulation of iAβ accompanied by the oxidation of DJ-1 (Figure 10A), basal lysosomal signal (Figure 11A), and the aberrant accumulation of autosomes in the absence of CC3 (Figure 12A), compared to untreated WT ChLNs. Investigation revealed that neither EGCG nor TM alone or in combination affected any of the variables tested, including iAβ and oxDJ-1, lysosomal signaling, autophagosomes, or CC3 in wild-type ChLNs (Figure 10B–F, Figure 11B–E, Figure 12B–F). Conversely, both reagents alone or in combination were highly effective in reducing these variables in mutant ChLNs. As shown in Figure 10, EGCG, TM, and EGCG/TM reduced iAβ by −66% (Figure 10B), −85% (Figure 10C), and −76% (Figure 10D), respectively, in PSEN1 E280A ChLNs compared to untreated mutant ChLNs (Figure 10E). Similarly, EGCG, TM, or TM/EGCG reduced the levels of oxidized DJ-1 by −84% (Figure 10B–D) for all treatments in mutant cells compared to untreated mutant ChLNs (Figure 10F). Although EGCG and TM alone or in combination did not affect lysosome signals in wild-type and mutant ChLNs (Figure 11A–E), both reagents were able to reduce the aberrant accumulation of autophagosomes. As shown in Figure 12, EGCG, TM, or EGCG/TM reduced autophagosome accumulation in PSEN1 E280A ChLNs by −53% (Figure 12B), −29% (Figure 12C), and 91% (Figure 12D), respectively, compared to untreated mutant ChLNs (Figure 12E). Furthermore, the combination of EGCG and TM exhibited a significantly more pronounced effect in blunting the LC3-II signal compared to EGCG and TM administered individually (*p* < 0.001). In summary, the effects observed for EGCG and TM on both WT and mutant ChLNs were consistent when analyzed by fluorescence microscopy (Figure 10G–P, Figure 11F–N and Figure 12G–P).

### 2.8. EGCG and TM Restore the Acetylcholine-Induced Ca^2+^ Influx Unresponsiveness in PSEN1 E280A ChLNs

**Figure 13:**
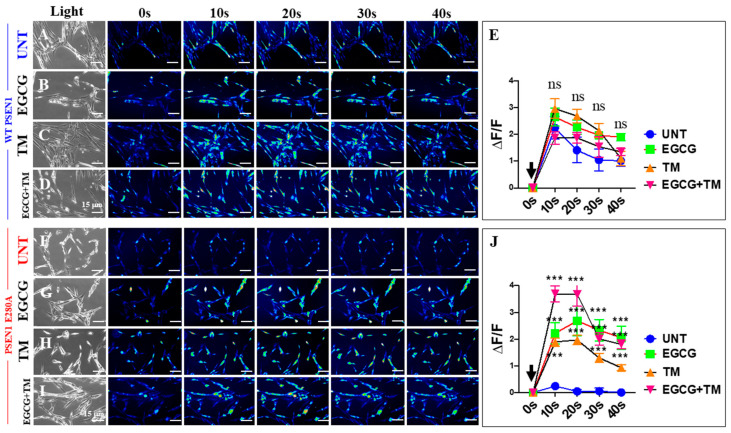
Fluorescence analysis of cholinergic-like neurons (ChLNs) acetylcholine (ACh)-mediated Ca^2+^ influx after EGCG and tramiprosate (TM) treatment. Both WT PSEN1 and E280A ChLNs were left untreated (UNT; (**A**,**F**)) or treated with 25 μM EGCG (**B**,**G**) and 50 μM TM (**C**,**H**) alone or in combination (**D**,**I**) for 7 days. Time-lapse images (0, 10, 20, 30, and 40 s) of Ca^2+^ fluorescence in ChLNs (*n* = 9, 20 imaged, N = 3 dishes) as a response to ACh treatment. ACh was puffed into the culture at 0 s (black arrow). Then, the Ca^2+^ fluorescence of cells was monitored at indicated times. Color contrast indicates fluorescence intensity: dark blue < light blue < green < yellow < red. (**E**) Normalized mean fluorescence signal (ΔF/F) ± SEM over time, indicating temporal cytoplasmic Ca^2+^ elevation in response to ACh treatment in WT PSEN1 ChLNs. (**J**) Normalized mean fluorescence signal (ΔF/F) ± SEM over time, indicating temporal cytoplasmic Ca^2+^ elevation in response to ACh treatment in PSEN1 E280A ChLNs. Significant values were determined by two-way ANOVA with a Tukey post hoc test; *** *p* < 0.001. n.s. = not significant. Image magnification 20×.

Previous studies have shown that PSEN1 E280A ChLNs are unresponsive to ACh-induced Ca^2+^ influx [41]. Therefore, we investigated whether EGCG and TM, either individually or in combination, could ameliorate the aberrant response of PSEN1 E280A to ACh stimuli. As shown in Figure 13, WT ChLNs enhanced the transient Ca^2+^ influx response to ACh (mean fluorescence change (ΔF/F) = 2.25 ± 0.42 and mean duration of 10 s each (*n* = 20 ChLN cells imaged, N = 3 dishes)) (Figure 13A,E). Conversely, PSEN1 E280A ChLNs showed no response to ACh stimulation (mean fluorescence change (ΔF/F) = 0.24 ± 0.09, mean duration of 10 s each (*n* = 20 ChLN cells imaged, N = 3 dishes)) (Figure 13F,J). When exposed to ACh and reagents, WT and PSEN1 E280A ChLNs showed different responses to ACh stimuli and reagents. No statistical difference was observed between the ACh-induced Ca^2+^ influx in the presence of EGCG (Figure 13B, ΔF/F = 2.65 ± 0.42) or TM (Figure 13C, ΔF/F = 2.97 ± 0.36) and EGCG plus TM (Figure 13D, ΔF/F = 1.87 ± 0.24) and untreated WT ChLNs (mean duration of 10 s each, Figure 13E) in intergroup and intragroup comparisons. Notably, the co-treatment of ACh with EGCG (Figure 13G, ΔF/F = 2.22 ± 0.39), TM (Figure 13H, ΔF/F = 1.89 ± 0.15), and the combination of EGCG and TM (Figure 13I, ΔF/F = 3.68 ± 0.29) dramatically increased the Ca^2+^ ion influx in PSEN1 E280A compared to untreated mutant ChLNs (mean duration of 10 s each, Figure 13J), albeit to different extents. The strongest effect on ACh-induced Ca^2+^ influx was observed with the co-treatment of combined EGCG and TM (Figure 13J), followed by a similar effect on cells treated with EGCG or TM alone (Figure 13J).

## 3. Discussion

To the best of our knowledge, this study is the first to investigate the development of PSEN1 E280A MenSCs-derived ChLNs in association with the kinetics of the accumulation of iAβ the ALP and other pathological markers up to day 7 to better understand the pathogenic mechanism of the development, progression, and resolution of FAD [59]. The study showed that PSEN1 E280A delayed the proliferative capacity of MenSCs without affecting their transdifferentiation into ChLNs. Consequently, a decrease in the proliferative marker Ki-67 and the disappearance of the mesenchymal marker CD73 were detected in MenSCs during their transition to PSEN1 E280A ChLNs. Notably, the expression levels of NeuN and ChAT remained unchanged in both WT and mutant ChLNs. These observations suggest that the PSEN1 E280A mutation does not affect the development of MenSCs into ChLNs. Notably, this study provides novel evidence that PSEN1 E280A induces a steady, time-dependent increase in the accumulation of iAβ from MenSCs to ChLNs, suggesting that the PSEN1 aspartyl protease is active in MenSCs and that its loss of function caused by mutations, e.g., E280A, impairs enzymatic activity during early cell development and differentiation [60,61].

Remarkably, we observed a concomitant accumulation of iAβ and oxidized DJ-1 (i.e., DJ-1C106-SO_3_) exclusively in mutant ChLNs at day 7 [41]. This observation suggests that iAβ requires an intermediate or organelle to generate reactive oxygen species (ROS) and OS. Given the established role of Aβ in inhibiting complex I, III, or IV of the electron transfer pathway of the electron transport chain (ETC), resulting in increased ROS production [62,63], it is conceivable that Aβ interacts with fully differentiated mitochondria at day 7 [64]. However, further studies are needed to substantiate this hypothesis. Notably, PSEN1 E280A induced a steady, time-dependent increase in the accumulation of autophagosomes, but not lysosomes, in the absence of cleaved caspase 3 (CC3) in mutant ChLNs. Taken together, these observations suggest that iAβ-induced OS coincides with a fully mature state of PSEN1 E280A ChLNs, as represented by the maximal expression of Neu and ChAT and the maximal accumulation of autophagosomes (LC3-II-positive cells), but not cell death (CC3-negative cells), in mutant neuron-like cells. Thus, it was found that the PSEN1 E280A mutation induces aberrant ALP and OS in ChLNs by day 7 of MenSC development but in the absence of apoptosis. However, the absence of differences in the cellular behavior of lysosomes between wild-type (WT) and mutant ChLNs suggests a disruption of the autophagy–lysosome system at the level of autophagosomes rather than lysosomal structures [10,11]. Regardless of the underlying mechanism, our results demonstrate that the early accumulation of iAβ inhibits ALP and induces OS in ChLNs harboring the PSEN1 E280A mutation.

It has been postulated that the use of RAP may prove beneficial in both the pre-symptomatic and early stages of Alzheimer’s disease, although the heterogeneity of experimental models (e.g., mouse and cell lines) and the different methodologies employed across studies have hindered the consistency of findings. These include the route of administration, dosage, timing, frequency, and availability of targeting vehicles. Together, these factors have hindered the use of RAP in AD patients [23,65]. In this study, we demonstrate for the first time that RAP, an mTOR inhibitor that enhances autophagy in neurons [66], did not attenuate PSEN1 E280A-induced pathological features, including Aβ, OS, and ALP, in PSEN1 E280A ChLNs. Our results are supported by several observations. First, RAP significantly increased iAβ and DJ-1C106-SO_3_ accumulation in WT cells but not in mutant ChLNs. Second, RAP increased lysosomes and the accumulation of autophagosomes in WT cells, whereas the AL system remained unperturbed in mutant ChLNs. Third, BAF, used as a control, led to a significant increase in iA and DJ-1C106-SO_3_ in WT cells, while maintaining stability for both markers in mutant ChLNs. These observations suggest that the early accumulation of iAβ blocks the fusion of autophagosomes and lysosomes, thereby blocking the process of autophagic degradation, without affecting cell survival (i.e., the cell death marker was CC3-negative) in PSEN1 E280A ChLNs. Our results suggest that RAP may not be neuroprotective at the early stages of FAD in PSEN1 E280A ChLNs. This contention is further supported by the ongoing controversy in preclinical research on RAP ([65] and this work) and the inconclusive results of ongoing clinical trials (e.g., ClinicalTrials.gov. ID NCT06022068 [67], NCT04200911). Until these issues are resolved, the use of RAP for the treatment of FAD may be considered [68].

It is hypothesized that the inhibition of BACE [69,70] and γ-secretase [28,29] could serve as promising early pharmacological strategies to reduce or block the generation of Aβ. We find that VER [27,28] and CE [25] significantly reduced iAβ at the earliest stages of ChLNs development, but the OS-associated marker DJ-1C106-SO_3_ was unaffected in PSEN1 E280A ChLNs. These observations suggest that, although VER and CE, as expected, are effective in targeting BACE and heterozygous γ-secretase in PSEN1 E280A, both reagents may disrupt other metabolic processes that generate OS. Remarkably, VER and CE induced a substantial accumulation of iAβ in WT ChLNs, resulting from the obstruction of normal (homozygous) BACE1 and normal (homozygous) γ-secretase, thereby mirroring the pathological characteristics of untreated PSEN1 E280A ChLNs. This assumption is further supported by the observation that VER and CE did not affect ALP in PSEN1 E280A ChLNs; however, both compounds induced autophagosome accumulation in WT ChLNs to an extent comparable to that observed in untreated or treated PSEN1 E280A. Therefore, it can be concluded that the use of VER and CE during the early stages of mutant ChLN development may prove beneficial in reducing iAβ. However, their application resulted in the unregulated expression of other processes, including OS and/or γ-secretase-independent effects on the autophagy lysosomal system [10] and signaling pathways [71]. Given the potential for VER and CE to induce off-target effects, our findings are consistent with the hypothesis that the development of BACE1 [70] and γ-secretase [29] inhibitors should prioritize the creation of BACE1/γ-secretase inhibitors with enhanced specificity and reduced side effects (e.g., see [72]).

Our laboratory has previously demonstrated that either EGCG or TM protects PSEN1 E280A or I416T ChLNs against Aβ-induced OS [47,48,51] through anti-amyloid [49,50,73,74] and/or antioxidant mechanisms [75]. In this study, we present novel findings showing that EGCG and TM, either alone or in combination, significantly reduced the early accumulation of iAβ and the concomitant oxidation of the stress sensor protein DJ-1 in PSEN1 E280A ChLNs. This effect was most likely due to the previously reported anti-amyloid and antioxidant mechanisms. Notably, the compounds, either alone or in combination, did not induce adverse effects in WT ChLNs, as evidenced by the minimal appearance of iAβ, DJ-1 oxidation, and CC3 markers. Furthermore, our results show that EGCG and TM, when administered alone or in combination, exhibited a modest reduction in the accumulation of autophagosomes. However, the combination of EGCG and TM resulted in a significant elimination of these abnormal markers, approaching levels comparable to those observed in untreated WT ChLNs. How does EGCG, TM, or the combination EGCG/TM increase autophagy flux? One possible explanation is that the decrease in autophagosomes is a consequence of the anti-amyloid activity of both compounds. Alternatively, EGCG may directly target iAβ aggregates and promote their lysosomal degradation [76] by increasing lysosomal fusion with autophagosomes and final disposal in the autolysosome. The mechanism by which TM increases autophagy flux and thereby decreases iAβ requires further investigation. We hypothesize that TM may increase autophagy flux through direct (by targeting iAβ aggregates through an unknown mechanism and promoting their lysosomal degradation) or indirect (through antioxidant and anti-amyloid mechanisms) actions. Interestingly, EGCG and TM, either as monotherapy or in combination, demonstrated efficacy in restoring the impaired ACh-induced Ca^2+^ influx in PSEN1 E280A ChLNs. A comprehensive analysis reveals that both EGCG and TM serve as effective preventive measures against PSEN1 E280A-induced OS and ALP dysfunction, as well as against the eAβ-induced blockade of ACh-responsive receptors, such as α-7nAChRs [77,78], which are involved in the intracellular accumulation of Aβ [79,80]. Taken together, the available data suggest that combined therapy rather than monotherapy approaches targeting autophagy together with antioxidants and/or anti-amyloids will be more favorable to combat the early aggregation of iAβ in mutant ChLNs, thereby improving the survival of affected ChLNs [81] and, more generally, the management of FAD [82]. Of note, recent clinical trials with a TM-derived prodrug known as ALZ-801/valiltramiprosate [83] have shown encouraging results in the treatment of AD [50,57]. Although clinical trials with EGCG have not yet advanced in AD, the available data suggest that a combination of TM (or its derivative ALZ-81) and EGCG (or its derivatives) [84] administered early to asymptomatic PSEN1 E280A familial early-onset patients [85,86,87] might be a promising therapeutic approach for the clearance of iAβ in the affected ChLNs. This strategy, if implemented at the earliest stages of FAD, could prevent the accumulation of neurotoxic iAβ and the aggregation of eAβ into plaques, potentially slowing disease progression. Furthermore, our data suggest that combined treatment (e.g., EGCG/TM) initiated at the earliest stage of life may be beneficial in young (or in children) carriers of the PSEN1 E280A variant [88].

## 4. Materials and Methods

### 4.1. Isolation and Characterization of Mesenchymal Stromal Cells Derived from Human Menstrual Blood (MenSCs)

Menstrual blood (MenB) samples were collected from a healthy female and a female carrier of the PSEN1 E280A mutation, aged 29 years (MenSC-CNT1) and 20 years (MenSC-E280A1). The donors provided signed informed consent approved by the Ethics Committee of the Sede de Investigación Universitaria (SIU), University of Antioquia, Medellín, Colombia. MenB was collected by cup collection (10–15 mL) during the first 3 days of menses. Briefly, menstrual blood samples were delivered to the laboratory and mixed with an equal volume of phosphate-buffered saline (PBS) containing 1 mM ethylenediaminetetraacetic acid (EDTA), with 100 U/mL penicillin/streptomycin and 0.25 mg/mL amphotericin B, and subjected to cell lysis or standard Ficoll procedures within 24 h, as previously described [89]. After centrifugation, the cell suspension in a buffy coat (7.7 × 10^6^ ± 2 × 10^6^ cells, *n* = 2) was transferred to a new tube, washed twice in PBS, and resuspended in growth medium (low-glucose DMEM medium supplemented with 10% FBS (Gibco, New York, NY, USA), 100 U/mL penicillin/streptomycin, and 0.25 mg/mL Amphotericin B) and seeded into 25 cm^2^ plastic cell culture flasks at 37 °C with 5% humidified CO2. The medium was changed every 3 days, leaving the adherent cells growing in clusters as fibroblastic cells. When the cells reached 80–90% confluence (P0), they were detached with 0.25% trypsin/1 mM EDTA and subcultured in new flasks at a 1:3 ratio.

### 4.2. Cholinergic-like Neuron (ChLN) Differentiation

ChLN differentiation was performed according to [40]. WT PSEN1 or PSEN1 E280A MenSCs were seeded at 1–1.5 × 10^4^ cells/cm^2^ in laminin-treated culture plates or flasks in regular culture medium for 24 h. The medium was then removed, and the cells were incubated in cholinergic differentiation medium (cholinergic N-Run medium, hereafter Ch-N-Rm) containing DMEM/F-12 media 1:1 nutrient mixture (Gibco cat# 10565018, NY, USA), 10 ng/mL basic fibroblast growth factor (bFGF) recombinant human protein (Gibco cat# 13256029), 50 μg/mL sodium heparin (Hep, Sigma-Aldrich cat# H3393, Sigma-Aldrich, St. Louis, MO, USA), 0.5 μM all-trans retinoic acid, 50 ng/mL sonic hedgehog peptide (SHH, Sigma-Aldrich cat# SRP3156, St. Louis, MO, USA), and 1% FBS at 37 °C for 0, 1, 3, 5, and 7 days.

### 4.3. Treatment Conditions

WT PSEN1 or PSEN1 E280A MenSCs were treated with or without rapamycin (RAP; 1 nM, Cat No. 53123-88-9, Sigma-Aldrich, St. Louis, MO, USA), bafilomycin A1 (BAF; 1 nM, Cat No. B1793, Sigma-Aldrich, St. Louis, MO, USA), verubecestat (VER; 500 pM Cat No. S8564, Selleck Chemical, Houston, TX, USA), γ-secretase inhibitor XXI, compound E (CE; 500 pM Cat No. 565790, Sigma-Aldrich, St. Louis, MO, USA), (−)-epigallocatechin-3-gallate (EGCG; 25 μM, Cat No. E4143, Sigma-Aldrich, St. Louis, MO, USA), or tramiprosate (TM; 50 μM Abcam Cat No. AB141116, Cambridge, MA, USA) for 7 days with medium refreshment every 3 days.

### 4.4. Immunofluorescence Analysis by Flow Cytometry

ChLNs were incubated in Ch-N-Rm for 0, 1, 3, 5, and 7 days alone or with inhibitors or antioxidants, as described above. The cells (1 × 10^5^) were then detached, washed twice with PBS (pH 7.2), and stored overnight at −20 °C in 95% ethanol. For the analysis of differentiation, neuronal, Alzheimer’s disease, oxidative stress, autophagy, and cell death-related markers, cells treated under different conditions were washed and incubated with 10% bovine serum albumin (BSA) blocking solution for 30 min. For the analysis of neuronal Alzheimer’s disease, oxidative stress, autophagy, and cell death-related markers, cells were incubated overnight with primary antibodies against Ki-67 (rabbit; abcam; cat. #ab15580, Cambridge, UK), CD73 (mouse; Elabscience; cat. #E-AB-F1242E, Houston, TX, USA), NeuN (rabbit; abcam; cat. #ab177487, Cambridge, UK), ChAT (ChAT, Millipore; cat. #AB144 P, Burlington, MA, USA), protein amyloid β1-42 (mouse, Millipore; cat. #MABN639, Burlington, MA, USA), oxidized DJ-1 (oxDJ-1; spanning residue C106 of human PARK7/DJ1; oxidized to cysteine sulfonic acid (SO_3_); rabbit, abcam; cat. #ab169520, Cambridge, UK), and LC3-II (rabbit, Novusbio; cat. #NB100-2220, Centennial, Colorado, USA) or 1X CellEvent Caspase3/7 Detection Reagent (Invitrogen, cat. # C10423, Carlsbad, CA, USA). Primary antibodies were prepared at a final concentration of 1:200. After thorough washing, cells were incubated with secondary fluorescent antibodies (DyLight 488 and 594 horse anti-rabbit, goat, and mouse; cat DI 1094, DI 3088, and DI 2488, respectively) at 1:500. Finally, the cells were washed and resuspended in PBS for analysis on an LSR-Fortessa (BD Biosciences, Franklin Lakes, NJ, USA). Ten thousand events were acquired, and acquisition analysis was performed using FlowJo version 7.6.2 (https://www.flowjo.com/previous-versions-flowjo, accessed on 10 March 2025) data analysis software. Positive staining was defined as a fluorescence emission exceeding the level of the population stained with the negative control.

### 4.5. Immunofluorescence Analysis by Fluorescent Microscopy

ChLNs were incubated in Ch-N-Rm for 0, 1, 3, 5, and 7 days alone or with inhibitors or antioxidants, as described above. The cells were then fixed with 4% formaldehyde for 20 min, followed by simultaneous permeabilization with Triton X-100 (0.1%) and blocking with 10% bovine serum albumin (BSA). For the analysis of neuronal Alzheimer’s disease, oxidative stress, autophagy, and cell death-related markers, cells were incubated overnight with primary antibodies against Ki-67, CD73, NeuN, ChAT, protein amyloid β1-42, oxidized DJ-1 (oxDJ-1C106) spanning residue C106 of human PARK7/DJ1 oxidized to cysteine sulfonic acid (SO_3_), and LC3-II or 1X CellEvent Caspase3/7 Detection Reagent (Invitrogen, cat. # No. C10423, ) (see above). Primary antibodies were prepared at a final concentration of 1:200. After thorough washing, cells were incubated with secondary fluorescent antibodies (DyLight 488 and 594 horse anti-rabbit, goat, and mouse, cat DI 1094, DI 3088, and DI 2488, respectively) at 1:500. Cell nuclei were stained with 1 μM Hoechst 33342 (Life Technologies, Carlsbad, CA, USA). Fluorescence microscopy images were captured using a Zeiss Axio Vert.A1 fluorescence microscope equipped with a Zeiss AxioCam Cm1 (Zeiss Wöhlk-Contact-Linsfluoreen, Gmbh, Schoenkirchen, Germany).

### 4.6. Live Fluorescence Analysis

ChLNs were incubated in Ch-N-Rm for 0, 1, 3, 5, and 7 days alone or with inhibitors or antioxidants, as described above. ChLNs were incubated in Ch-N-Rm for 0, 1, 3, 5, and 7 days alone or with inhibitor or antioxidants, according to the description above. Then, cells were incubated with the cell-permeable, nonfixable, green, fluorescent dye LysoTracker Green DND-26 (50 nM, cat #L7526, Thermo Fisher Scientific, Waltham, MA, USA) at 37 °C in the dark for 30 min. The nuclei were stained with Hoechst 33342 (1 µM, Life Technologies). Then, the cells were washed, and probe fluorescence was determined by flow cytometry using a BD LSRFortessa II flow cytometer (BD Biosciences, Franklin Lakes, NJ, USA) or fluorescent microscopy using a Zeiss Axio Vert.A1 Fluorescence Microscope equipped with a Zeiss AxioCam Cm1 (Zeiss Wöhlk-Contact-Linsfluoreen, Gmbh, Schoenkirchen, Germany). The experiment was conducted three times, and 10,000 events were acquired for analysis. Flow cytometry analysis was performed by selecting the FL-1 channel.

### 4.7. Intracellular Calcium Imaging

Changes in intracellular calcium (Ca^2+^) concentration induced by cholinergic stimulation were assessed according to previous reports [90,91] with minor modifications. The fluorescent dye Fluo-3 (Fluo-3 AM; Thermo Fisher Scientific, cat: F1242) was used for measurement. The dye was dissolved in DMSO (1 mM) to form a stock solution. Before experiments, the stock solution was diluted in neuronal buffer solution (NBS buffer: 137 mM NaCl, 5 mM KCl, 2.5 mM CaCl_2_, 1 mM MgCl_2_, pH 7.3, and 22 mM glucose). The working concentration of dye was 2 μM. The WT and PSEN1 E280A ChLNs were incubated with the dye-containing NBS for 30 min at 37 °C and then washed five times. Intracellular Ca^2+^ transients were elicited by acetylcholine (1 mM final concentration). Measurements were performed with the 20× objective of the microscope. Several regions of interest (ROIs) were defined in the field of view of the camera. One of the ROIs was cell-free, and the fluorescence intensity measured here was considered as background fluorescence (F_bg_). The time dependence of the fluorescence emission was recorded, and the fluorescence intensities (hence the Ca^2+^ levels) were represented by pseudocolors. To calculate the changes in average Ca^2+^-related fluorescence intensities, the Fbg value was determined from the cell-free ROI, and then, the resting fluorescence intensities (F_rest_) of the cell-containing ROIs were obtained as the average of the points recorded during a period of 10 s before the addition of acetylcholine. The peaks of the fluorescence transients were found by calculating the average of four consecutive points and identifying the points that gave the highest average value (F_max_). The amplitudes of the Ca^2+^-induced fluorescence transients were expressed relative to the resting fluorescence (ΔF/F) and calculated using the following formula: ΔF/F = (F_max_ − F_rest_)/(F_rest_ − F_bg_). ImageJ software version 1.54p (http://imagej.nih.gov/ij/ (accessed on 10 January 2025)) was used to calculate fluorescence intensities.

### 4.8. Characterization of Lysosomal Complexity

To analyze lysosomal complexity [92,93], cells were incubated with the cell-permeable, non-fixable, green fluorescent dye LysoTracker Green DND-26 (50 nM, cat #L7526, Thermo Fisher Scientific, Waltham, MA, USA) for 30 min at 37 °C. The cells were then washed, and LysoTracker fluorescence was determined by flow cytometry using a BD LSR-Fortessa II flow cytometer (BD Biosciences, Franklin Lakes, NJ, USA) or by fluorescence microscopy using a Zeiss Axio Vert.A1 fluorescence microscope equipped with a Zeiss AxioCam Cm1. The experiment was performed three times, and 20,000 events were acquired for analysis. Flow cytometry analysis for the LysoTracker was performed by selecting all cells with LysoTracker reactivity (>99%) in the FL-1 channel. Quantitative data and figures were obtained using FlowJo 7.6.2 data analysis software (BD Biosciences, Franklin Lakes, NJ, USA).

### 4.9. Photomicrography and Image Analysis

Light microscopy and fluorescence microscopy images were captured using a Zeiss Axiostart 50 fluorescence microscope equipped with a Zeiss AxioCam Cm1 and (Zeiss Wöhlk-Contact-Linsfluoreen, Gmbh, Schoenkirchen, Germany). Fluorescence images were analyzed using ImageJ software version 1.54p (http://imagej.nih.gov/ij/ (accessed on 10 January 2025)). The images were converted to 8-bit images, and the background was subtracted. The cellular regions of interest (ROIs) were drawn around the nucleus (for transcription factors and apoptosis effectors) or over all cells (for cytoplasmic probes), and fluorescence intensity was then determined using the same threshold for cells in control and treatment conditions. The mean fluorescence intensity (MFI) was obtained by normalizing the total fluorescence to the number of nuclei.

### 4.10. Data Analysis

In this experimental design, a vial of MenSCs was thawed and cultured, and the cell suspension was pipetted at a standardized cell density of 2.6 × 10^4^ cells/cm^2^ into different wells of a 24-well plate. Cells (i.e., the biological and observational unit [94]) were randomized to wells by simple randomization (sampling without replacement method), and then, wells (i.e., the experimental units) were randomized to treatments by a similar method. Experiments were conducted in triplicate wells. Data from individual replicate wells were averaged to yield a value of *n* = 1 for that experiment, and this was repeated three times, blinded to the experimenter and/or flow cytometer analyst, for a final value of *n* = 3 [94]. Based on assumptions that the data from the experimental unit (i.e., well) met the independence of observations, that the dependent variable was normally distributed in each treatment group (Shapiro–Wilk test), and that there was homogeneity of variances (Levene’s test), statistical significance was determined by a one-way analysis of variance (ANOVA) followed by Tukey’s post hoc comparison calculated using GraphPad Prism 5.0 software. Differences between groups were considered significant only when the *p*-value was <0.05 (*), <0.001 (**), and <0.001 (***). All data are presented as mean ± S.D.

## 5. Conclusions

**Figure 14:**
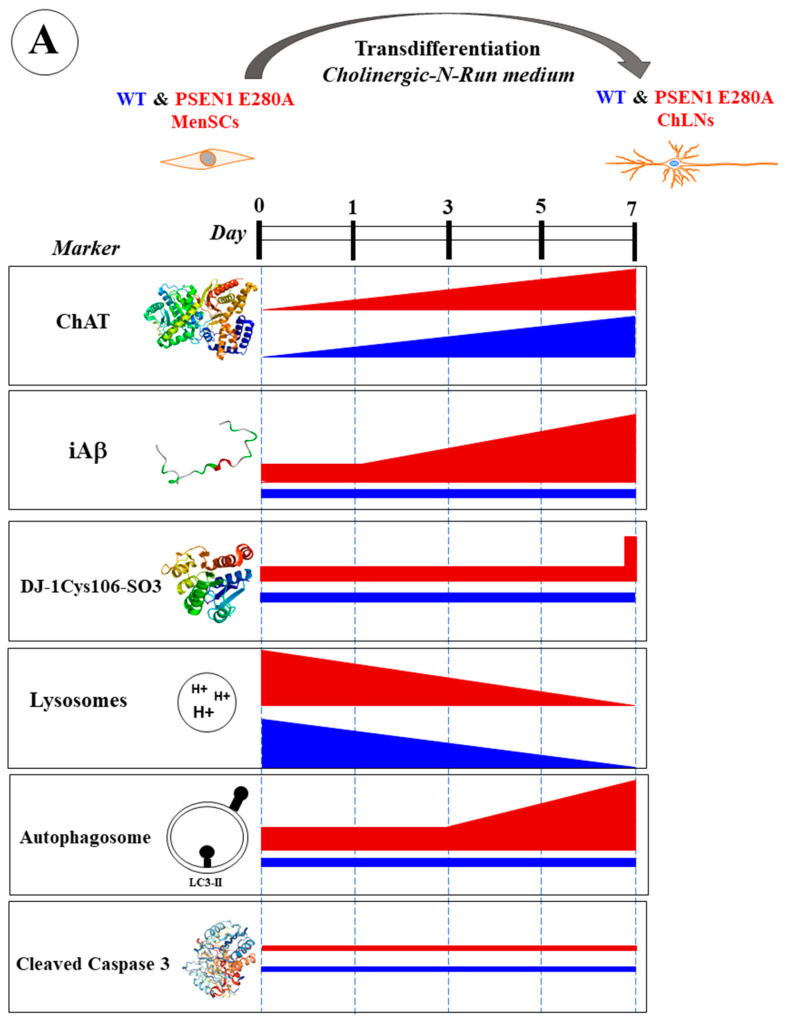

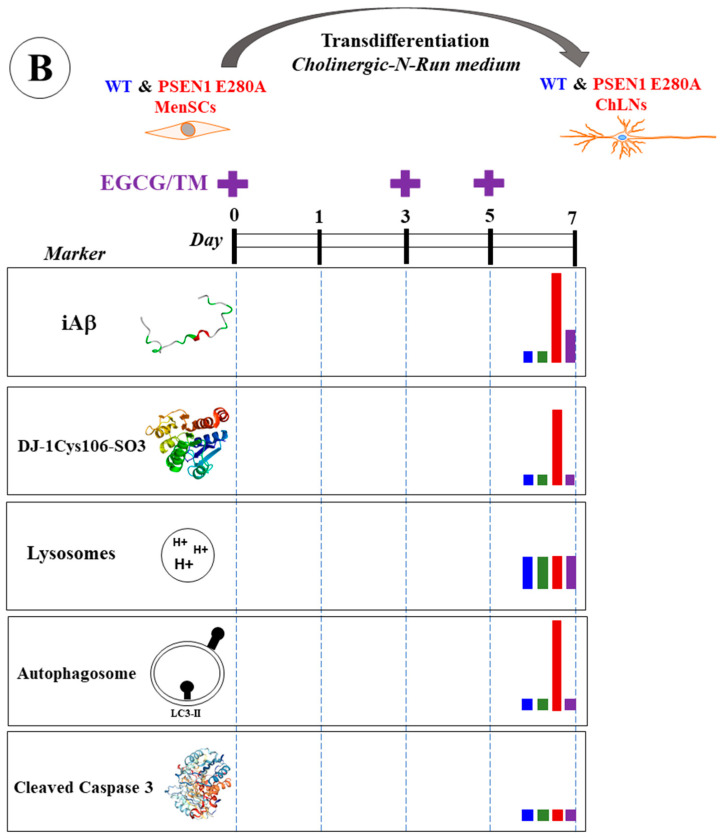
(**A**) Schematic representation of the transdifferentiation of wild-type (WT) and PSEN1 E280A mesenchymal stromal cells (MenSCs) into cholinergic-like neurons (ChLNs) cultured in cholinergic-N-Run medium up to day 7 and major neuropathologic markers. (**B**) Schematic representation of WT and PSEN1 E280A MenSCs-derived ChLNs treated with a combination of epigallocatechin-3-gallate (EGCG) and tramiprosate (TM) at days 0, 3, and 5. Evaluation of markers were performed at day 7. Blue color bar represents untreated WT ChLNs, green color bar represents WT treated with combined EGCG/TM, red color bar represents untreated PSEN1 E280A, and purple color bar represents PSEN 1 E280A cells treated with combined EGCG/TM.

Our results show that Aβ begins to accumulate intracellularly at an early stage of transdifferentiation of PSEN1 E280A MenSCs into ChLNs from day 0, corresponding to the MenSCs stage, to day 7 in mutant ChLNs (Figure 14A). Mutant ChLNs exhibited an increase in OS at day 7, as well as an aberrant accumulation of autophagosomes starting at day 3 of cell transdifferentiation. Interestingly, LysoTracker^®^-positive low pH lysosomes and the cell death marker CC3 were barely or not detected in WT or PSEN1 E280A ChLNs (Figure 14A). The combination of EGCG and TM was found to almost completely reverse the pathological phenotype induced by PSEN1 E280A in ChLNs (Figure 14B). These findings may provide valuable information to confirm or exclude the therapeutic role of EGCG and TM, ideally in young children carrying the PSEN 1 E280A mutation, in FAD patients. However, the conclusions drawn from our present investigation need to be verified by additional studies with larger sample sizes, as the results were based on MenSCs from a single WT and PSEN1 E280A menstrual blood sample.

## Data Availability

All datasets generated for this study are included in the manuscript.
